# A multi-agent large language model (LLM) platform for structured medical anamnesis in outpatient care

**DOI:** 10.3389/fdgth.2026.1882397

**Published:** 2026-09-30

**Authors:** Elena Zaitseva, Simon Ochotnicky, Miriam Fandakova, Vitaly Levashenko, Jan Rabcan

**Affiliations:** Faculty of Management Science and Informatics, University of Zilina, Zilina, Slovakia

**Keywords:** clinical decision support, large language models, medical informatics, outpatient care, patient-generated health data, structured medical anamnesis

## Abstract

**Introduction:**

Medical anamnesis is fundamental to clinical decision-making, yet outpatient consultations often provide limited time for systematic information collection. This study investigates whether structured anamnesis acquisition can be redistributed to a pre-consultation phase using a framework-driven large language model (LLM) platform.

**Methods:**

We developed Symptio, a proof-of-concept multi-agent LLM platform that operationalises the SOCRATES, Review of Systems, and SAMPLE frameworks as machine-interpretable constraints within a human-in-the-loop workflow. The system was evaluated in 100 simulated outpatient scenarios. Generated anamneses were compared with reference-standard representations using structural agreement and field-level performance metrics.

**Results:**

Full structural agreement across all three frameworks was achieved in 87% of scenarios. Framework-specific agreement reached 96% for SOCRATES, 88% for Review of Systems, and 93% for SAMPLE. Micro-averaged precision, recall, and F1-score were 99.04%, 98.54%, and 98.79%, respectively. Although no high-severity errors were identified, 58 field-level discrepancies, including 28 medium-severity errors, were observed, supporting the need for physician review of all outputs.

**Discussion:**

The findings demonstrate the technical feasibility of acquiring structured anamnesis in a framework-driven manner within a process-oriented multi-agent LLM architecture under controlled conditions. The proposed approach enables the relocation of routine information collection to a pre-consultation phase while preserving physician authority through human-in-the-loop validation. Further studies are required to evaluate clinical safety, workflow impact, and effectiveness in real-world settings.

## 1 Introduction

Medical anamnesis remains one of the most information-dense components of clinical decision-making and continues to play a central role in diagnosis, management, and patient safety. Classical studies have consistently shown that a substantial proportion of correct diagnoses can be established primarily through patient history, even before physical examination or diagnostic testing (1, 2). Despite this importance, history taking in outpatient care is frequently performed under strict time constraints and in parallel with extensive documentation requirements imposed by modern Electronic Health Record (EHR) systems. Clinicians are therefore required to simultaneously listen, interpret, structure, and transcribe patient narratives during the encounter, contributing to cognitive load, workflow fragmentation, and clinician burnout. This reframing moves routine anamnesis preparation outside the time–critical physician–patient encounter.

From a clinical workflow perspective, outpatient encounters exhibit a structural imbalance: while physicians experience time scarcity during consultations, patients often spend substantial time waiting before being seen. This waiting-room interval represents an underutilised window for information acquisition. Previous work in clinical operations and access management has identified patient waiting time as a significant contributor to inefficiency and dissatisfaction, but has largely focused on scheduling optimisation rather than clinical data collection (3, 4). Reframing this interval as a preparatory phase for medical anamnesis opens an opportunity to redistribute routine information-gathering effort outside the time-critical physician–patient interaction.

A core difficulty in anamnesis collection lies in the mismatch between patient narratives and clinical reasoning. Patients naturally describe symptoms in unstructured, subjective, and often incomplete terms, whereas clinicians require information organised along specific diagnostic dimensions. To address this gap, medical education and clinical practice have long relied on structured frameworks such as SOCRATES for symptom characterisation (5), Review of Systems (ROS) for systematic screening across organ systems (6), and SAMPLE for capturing essential contextual history (7). These frameworks are deeply embedded in clinical cognition but are normally applied tacitly and verbally by trained clinicians rather than encoded explicitly in information systems (Table 1). These frameworks are also used in the developed proof-of-concept prototype platform Symptio.

**Table 1:** Clinical frameworks.

Framework	Clinical purpose	Operational role in Symptio
SOCRATES	Pain characterisation: site, onset, character, radiation, associations, time course, exacerbating or relieving factors, and severity.	Guides targeted follow-up questions and fills symptom-specific fields.
ROS	Screening across organ systems to reduce tunnel vision and detect associated symptoms or red flags.	Triggers contextual questions when the initial complaint may be incomplete.
SAMPLE	Rapid contextual history: symptoms, allergies, medication, past history, last intake, and events.	Adds patient background needed for safe interpretation and documentation.

Most contemporary EHR interfaces provide static forms or templates that partially reflect these frameworks, yet they remain poorly aligned with the natural flow of patient narratives (8). As a result, clinicians often reconstruct structured information mentally before entering it manually, duplicating effort and increasing the risk of omission. From an engineering standpoint, this process represents a knowledge-translation problem: clinically validated heuristics exist, but they are not systematically formalised into machine-interpretable logic capable of guiding patient-entered data. It is a process that can be effectively implemented using generative artificial intelligence, in particular, Large Language Models (LLMs) (8, 9). There are platforms integrated into EHR systems that increasingly employ speech recognition and generative models to draft clinical notes from physician–patient conversations, helping clinicians focus more on patient interaction (10). For example, Abridge is one such platform developed with Epic EHR (11).

LLMs have generated strong interest in their application to clinical documentation, summarisation, and decision support (9, 12, 13). Multiple studies and systematic reviews have demonstrated that LLM-based systems and ambient documentation tools can reduce documentation time and administrative burden, particularly when used as assistants rather than as autonomous decision-makers.

However, the use of LLMs in clinical settings must be meaningful. As is known, LLMs can produce hallucinations, i.e., plausible but unsupported statements that may be clinically misleading (14, 15). In documentation tasks, hallucinations may manifest as invented symptoms, incorrect temporal relationships, or misplaced clinical emphasis, posing risks to patient safety and medico-legal accountability. Consequently, current best practices emphasise human-in-the-loop designs, constrained prompting, and task decomposition to limit model autonomy and maintain clinician oversight (16, 17).

Despite rapid advances in LLM-based medical systems, the preceding analysis highlights a persistent gap between functional task optimisation and the management of clinical care processes. As discussed in Section 2, most existing solutions adopt an encounter-centric design, focusing on documentation, transcription, or information delivery within or immediately after the physician–patient interaction. These approaches, while effective in reducing administrative burden, rarely model the temporal structure of outpatient care or exploit established clinical history-taking frameworks to redistribute routine information collection beyond the time-critical consultation phase.

This study addresses this gap by presenting Symptio, a proof-of-concept medical information platform that adopts a process-oriented perspective on outpatient anamnesis collection. Rather than aiming to automate diagnosis or replace clinical judgment, Symptio operationalises clinically established anamnesis frameworks (Table 1) into machine-interpretable questioning logic and relocates structured information acquisition to a pre-consultation phase. Through a constrained, multi-agent LLM architecture, the platform is designed to support active patient participation in guided data collection while preserving physician authority through human-in-the-loop validation. The objective of this work is to investigate the technical feasibility of formalising established anamnesis methodologies (SOCRATES, Review of Systems, and SAMPLE) as machine-interpretable workflow constraints within a process-oriented multi-agent LLM architecture. The study focuses on the technical feasibility of structured information acquisition and transformation under controlled, simulated conditions rather than on clinical validation, diagnostic performance, healthcare outcomes, or evaluation in real patient care settings.

The contribution of this work does not lie in proposing a novel language-model architecture, benchmarking foundation models, or demonstrating clinical effectiveness. Rather, it lies in the formalisation of established anamnesis methodologies as explicit computational constraints and their integration into a workflow-oriented information acquisition pipeline. Accordingly, the proposed platform should be interpreted as a proof-of-concept exploration of workflow-oriented knowledge representation and process design rather than as a clinically validated decision-support system.

## 2 Related approaches and limitations of existing solutions

In contemporary medical applications, LLMs are being actively explored and deployed across multiple functional areas of healthcare, driven by their capacity to process unstructured natural language, summarise complex information, and generate clinically styled text (13, 14). Despite well-recognised limitations, including hallucinations, omissions, and inconsistent factual grounding, LLMs continue to gain adoption as assistive components when embedded within constrained, human-supervised workflows.

A major and widely adopted class of LLM-based systems focuses on clinical documentation and note generation, particularly through ambient medical scribe technologies (11, 18). These systems capture physician–patient conversations and reorganise them into structured clinical notes that clinicians can review and finalise. Commercial platforms such as Microsoft DAX Copilot, Abridge, and ModMed Scribe 2.0 exemplify this encounter-centric approach by integrating LLM-based transcription and summarisation directly into electronic health record environments. Empirical evidence and early clinical experience suggest that such systems can reduce documentation time and administrative burden, especially in outpatient care. However, they operate primarily during or immediately after the clinical encounter and do not fundamentally alter the structure or timing of anamnesis collection. Their core contribution lies in transcription and summarisation rather than in restructuring the flow of clinical information acquisition.

A second major direction involves clinical decision support and informational assistance, where LLMs are employed to provide context-specific medical information, guideline summaries, or explanatory support to clinicians and, in some cases, to patients through conversational interfaces (9, 19). Systems in this category include both research prototypes and early commercial offerings, such as agent-based or conversational platforms (e.g., ThinkAndor), which aim to mediate clinical knowledge exchange. While this direction demonstrates the potential of LLMs to augment clinical reasoning and communication, it also raises heightened safety and governance concerns. As a result, such systems typically rely on constrained prompting, *post hoc* validation, and explicit human oversight. In practice, their integration remains closely tied to physician-initiated queries and on-demand information retrieval rather than to proactive or workflow-driven data preparation.

Beyond these LLM-centric applications, substantial research attention has focused on patient-generated health data (PGHD) (8), including patient-entered information collected outside the clinical encounter via patient portals, pre-visit questionnaires, and remote monitoring platforms. PGHD-oriented approaches emphasise patient engagement and longitudinal data capture, particularly in chronic disease management. However, such data often arrive in heterogeneous or weakly structured formats and are not systematically aligned with classical anamnesis frameworks, potentially increasing clinician review burden rather than reducing it.

Despite their diversity, these approaches share a common limitation: LLMs are predominantly treated as isolated functional tools rather than as components embedded within the management of the clinical care process itself (10). Most existing systems adopt an encounter-centric design, optimising specific tasks, such as transcription, summarisation, explanation, or query-driven decision support, without explicitly modelling the temporal and organisational structure of outpatient care. In particular, they rarely address when clinical information is collected across different phases of care or leverage established clinical history-taking frameworks to redistribute routine information-gathering effort outside the time-critical physician–patient interaction.

The proposed platform Symptio has been developed in response to these limitations. By adopting a process-oriented perspective, this work examines LLMs not only as text generation tools but also as enablers of workflow reorganisation in medical care delivery. Anamnesis is treated as a structured information engineering task that can be partially relocated to a pre-consultation phase, provided that data collection is guided by clinically validated frameworks and remains subject to physician review. As a first step, the present study focuses on outpatient primary and general medical care, where anamnesis plays a central role, workflows are relatively standardised, and the potential impact of redistributing routine information gathering effort is particularly high.

To clarify this distinction, Table 2 summarises a process-oriented comparison between encounter-centric LLM-based systems and an alternative workflow-driven approach. Within this context, the proof-of-concept prototype platform Symptio is positioned not as another documentation tool, but as a medical information platform designed to reframe anamnesis collection within the outpatient care process. By explicitly aligning LLM-mediated interaction with clinically established history-taking frameworks and by relocating structured data acquisition to a pre-consultation phase, Symptio exemplifies how LLMs can be embedded into care-process reorganisation rather than applied solely to encounter-level tasks. Importantly, the present work focuses on the formalisation of established anamnesis methodologies as explicit workflow constraints rather than on optimisation of foundation-model performance itself.

**Table 2:** Process-oriented comparison of LLM-based systems for outpatient documentation and anamnesis.

Process Dimension	Encounter-Centric LLM Systems (e.g., DAX Copilot, Abridge, ModMed Scribe)	Symptio
Primary phase of use	During or immediately after the clinical encounter	Before the clinical encounter (waiting-room phase)
Care-process perspective	Encounter-centred	Care-process re-oriented
Primary objective	Documentation support and note drafting	Structured anamnesis preparation
Main input modality	Physician–patient conversation (audio/text)	Patient-entered narrative
Role of patient	Predominantly passive information source	Active participant in guided data collection
Clinical framework encoding	Implicit (templates, note sections)	Explicit (SOCRATES, ROS, SAMPLE)
LLM interaction logic	Free or weakly constrained summarisation	Constrained, framework-driven questioning
Hallucination risk mitigation	Post-generation physician review	Intended to mitigate hallucination risk through preventive constraints and agent separation
Physician role	Editor and verifier of drafted notes	Validator of pre-structured anamnesis
Workflow impact	Reduces post-encounter documentation burden	Designed to redistribute information acquisition across care phases
System positioning	Documentation aid	Medical information platform for anamnesis preparation

## 3 Materials and methods

### 3.1 System overview and design principles

Symptio is a proof-of-concept medical information platform designed to support structured medical anamnesis preparation in outpatient care through workflow re-engineering rather than encounter-level automation. Figure 1 provides a high-level comparison between the traditional outpatient anamnesis workflow and the process-oriented workflow implemented in Symptio.

**Figure 1:**
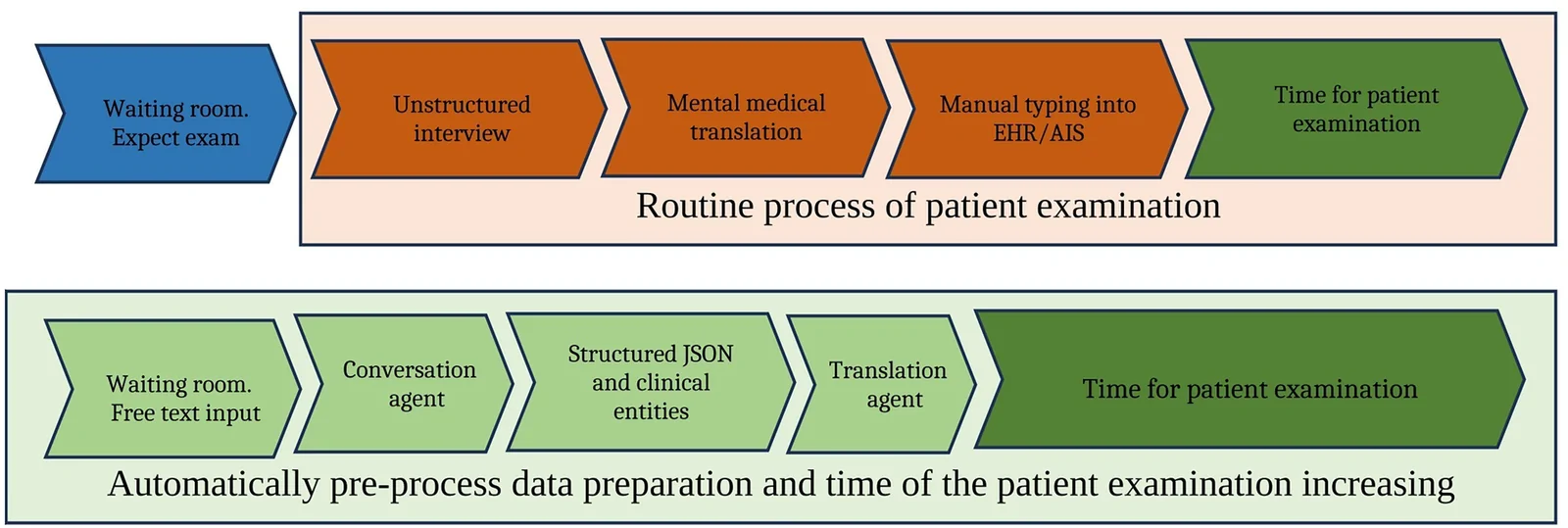
Process-level reframing of anamnesis collection from a sequential physician-driven workflow to an asynchronous patient-assisted workflow.

The system is explicitly conceived as a process-oriented, human-in-the-loop platform, in which large language models serve as assistive components for information organisation and transformation, rather than as autonomous diagnostic agents.

The core design objective of Symptio is to explore redistributing routine anamnesis data collection from the time-critical physician-patient encounter to a pre-consultation phase. This design choice is grounded in the temporal asymmetry of outpatient workflows, in which patients experience idle time before the consultation while clinicians operate under strict time constraints during the encounter. By relocating structured information acquisition upstream in the care process, the platform is intended to support future workflow models that improve consultation efficiency while preserving physician responsibility and human oversight.

From an architectural perspective, Symptio follows three foundational design principles. First, process primacy: the system is structured around the outpatient care workflow rather than around individual documentation or decision-support tasks. Anamnesis is treated as an information-engineering process with clearly defined input, transformation, and validation stages. Second, clinical framework formalisation: established history-taking schemas used in routine medical practice (SOCRATES, ROS, and SAMPLE in Table 1) are explicitly encoded into machine-interpretable questioning logic to guide data collection and organisation. Third, human clinical authority: all system outputs are designed to remain subject to physician review and validation, ensuring that clinical interpretation and decision-making are never delegated to the LLM components.

In this work, a multi-agent LLM architecture refers to a system design in which distinct language-model-based agents are assigned clearly separated roles within the clinical workflow, rather than operating through a single monolithic prompt. Each agent is constrained to a specific task scope (e.g., guided data collection or medical language transformation), enabling task decomposition, controlled interaction, and improved safety through role separation. This design follows established principles of agent-based systems and recent recommendations for the reliable deployment of LLMs in clinical contexts (13, 20, 21).

To operationalise these principles, Symptio employs a multi-agent LLM architecture comprising distinct patient-facing and physician-facing agents with clearly separated responsibilities. The patient-facing agent facilitates guided data collection from lay narratives prior to the encounter, while the physician-facing agent transforms the resulting structured state into a reviewable draft of standardised medical documentation. Crucially, the system enforces task decomposition and constrained interaction patterns to limit generative ambiguity and reduce the risk of generating unsupported content. These architectural choices were motivated by current recommendations for the safe and reliable use of LLMs in clinical support contexts.

The high-level cognitive transformation implemented by the platform is illustrated in Figure 2. The figure depicts the role of a specialised multi-agent pipeline through which lay patient narratives are progressively structured and translated into a clinically interpretable anamnesis representation. Importantly, the figure abstracts away implementation details and focuses on the functional separation of responsibilities between agents, reflecting the system's design principles rather than its technical execution.

**Figure 2:**
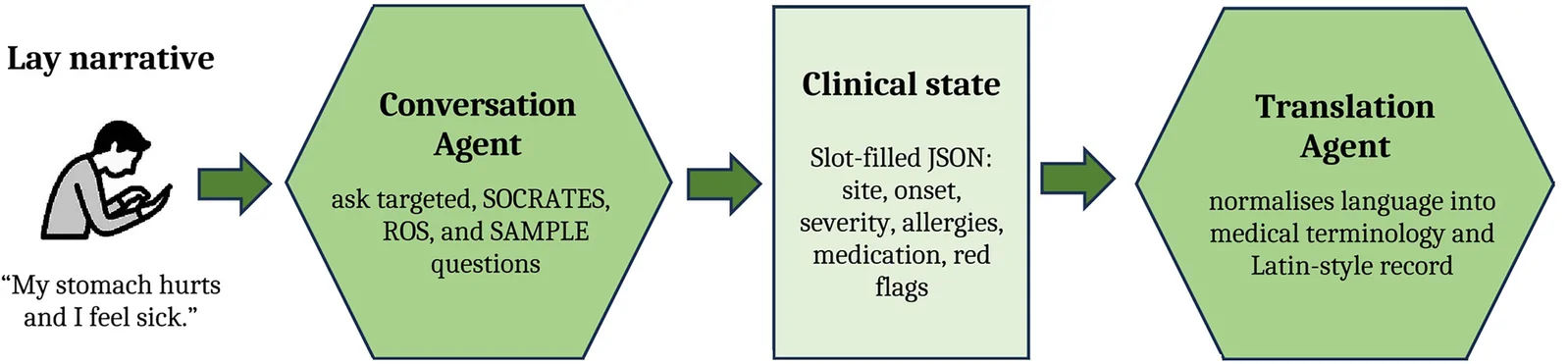
Multi-agent cognitive pipeline from lay patient narrative to structured clinical record.

The most important decision in Symptio is the separation between collection (Conversation Agent) and transformation (Translation Agent) (Figure 2). The Conversation Agent is patient-facing. Its behaviour is constrained by SOCRATES, Review of Systems, and SAMPLE. At each conversational turn, it produces a structured JSON output containing a patient-facing response and a state update rather than a diagnostic or clinical conclusion. The Translation Agent is physician-facing. It receives the structured state and produces a normalised medical record using appropriate terminology. In the evaluated prototype, both agents used the same supervised fine-tuned language model derived from the gpt-4.1-nano-2025-04-14 base model, while operating with different system prompts, contexts, and task-specific success criteria. The same fine-tuned model was used throughout all 100 evaluation scenarios. The bridge between agents is a structured JSON object. It contains the main complaint, SOCRATES fields where relevant, Review of Systems findings, SAMPLE context, red-flag indicators, confidence values, and metadata about the interaction. The JSON representation was designed to support persistence, auditability, and potential future integration with clinical information systems.

### 3.2 Process-oriented anamnesis workflow

The process-oriented anamnesis workflow implemented in Symptio is designed as a reorganisation of information acquisition within the outpatient care pathway rather than as an optimisation of encounter-level documentation tasks. A high-level comparison between the traditional encounter-centric workflow and the proposed process-oriented approach is illustrated in Figure 1. In the conventional model, anamnesis collection, interpretation, and documentation are performed synchronously during the physician–patient encounter, placing substantial cognitive and temporal demands on the clinician. In contrast, the workflow implemented in Symptio explicitly separates information acquisition from clinical interpretation and redistributes these activities across different phases of care.

In the proposed workflow, anamnesis preparation begins prior to the clinical encounter, typically during the waiting-room phase. At this stage, the patient engages with the system to provide a narrative description of symptoms, concerns, and relevant medical context. Rather than collecting free-text input alone, the workflow is designed to guide patient input through structured questioning aligned with established clinical anamnesis frameworks. This pre-consultation phase is explicitly non-diagnostic and does not involve clinical decision-making; its purpose is limited to structured information capture and organisation.

The structured information collected during the pre-consultation phase is subsequently transformed into a framework-aligned anamnesis representation intended for physician review. Importantly, this representation is intended to support, rather than replace, traditional clinical interaction. Physicians retain full authority to verify, amend, extend, or disregard any pre-collected information based on their professional judgment and direct patient interaction. By entering the encounter with a pre-structured anamnesis candidate, the physician can allocate a greater proportion of consultation time to clarification, physical examination, shared decision-making, and patient communication.

From a process-engineering perspective, the key distinction of this workflow lies in its explicit temporal realignment of tasks. Routine anamnesis data acquisition and structuring are relocated upstream in the care process, while clinical interpretation and decision-making remain firmly within the encounter itself. This separation reduces time pressure during the consultation without altering clinical responsibility boundaries. As shown in Figure 1, the proposed workflow concept remains sequential and auditable, with clearly defined transitions between patient-driven data entry, system-assisted structuring, and physician-led validation.

The process-oriented workflow implemented in Symptio is intentionally designed to be compatible with standard outpatient care routines and to require minimal behavioural change from clinicians. Patients are prompted to participate during an otherwise idle waiting period, while physicians primarily interact with the system through review and confirmation rather than active data entry. This design reflects the platform's central objective: leveraging LLM-based capabilities for structured language processing to support care-process reorganisation without introducing additional cognitive or safety burdens into clinical practice.

### 3.3 Multi-Agent LLM architecture

To operationalise the process-oriented anamnesis workflow described in Section 3.2, the Symptio platform employs a role-specialised multi-agent LLM architecture as a design mechanism for task decomposition and for limiting generative ambiguity. Rather than relying on a single, monolithic language-model prompt, the system assigns distinct agents clearly separated responsibilities, each operating under constrained task definitions and isolated contextual scopes.

In this work, a multi-agent architecture refers to a system design in which multiple LLM-based agents collaborate sequentially, with each agent performing a narrowly defined transformation step within the overall information pipeline. This design follows established principles of separation of concerns and reflects current recommendations for reliable and safety-aware deployment of LLMs in clinical support settings, which emphasise constrained generation and human oversight. A high-level representation of this cognitive pipeline is illustrated in Figure 2.

#### 3.3.1 Architectural rationale and agent decomposition

The adoption of a multi-agent architecture in Symptio was motivated by workflow separation, task decomposition, and controllability. The present study was not designed to evaluate whether a multi-agent architecture is superior to alternative implementations. Accordingly, the observed performance should be interpreted as evidence of the feasibility of the complete system rather than as evidence of the superiority of its individual components. First, task decomposition reduces the cognitive load imposed on any single model instance, limiting context confusion and unintended cross-task interference. Second, role isolation enables preventive mitigation of hallucination risks by restricting each agent's generative scope to well-defined operations, rather than relying on post-hoc correction.

In preliminary design iterations, monolithic prompting strategies, in which a single agent was tasked with patient interaction, clinical structuring, and translation of medical terminology, produced inconsistent outputs, including stylistic leakage between lay and professional language. To address these limitations, the final architecture separates the system into two cooperating agents, orchestrated by an external controller: a Conversation Agent and a Translation Agent. Each agent operates over the same underlying fine-tuned language model but is guided by task-specific system prompts and validation constraints.

#### 3.3.2 Patient facing conversation agent

The Conversation Agent is responsible exclusively for interacting with the patient during the pre-consultation phase. Its function is limited to the acquisition of guided anamnesis data and explicitly excludes diagnosis, interpretation, or recommendation. Patient input is accepted in unrestricted natural language, but the agent's questioning logic is constrained by algorithmically encoded clinical frameworks, specifically SOCRATES, ROS, and SAMPLE.

Internally, the agent maintains a structured state representation containing pre-defined fields corresponding to required anamnesis dimensions. During interaction, incoming patient responses are semantically analysed and used to populate this state. If critical fields remain unfilled (for example, symptom onset, duration, or associated factors), the agent autonomously generates targeted follow-up questions. The interaction terminates only once all framework-defined requirements have been satisfied. This deterministic completion criterion prevents premature summarisation and ensures consistency across patient sessions.

At each turn, the Conversation Agent returns a structured JSON turn object containing a patient-facing response and a structured state update. The patient-facing component is used for continued elicitation, while the validated state information is persisted by the orchestration layer and subsequently supplied to the Translation Agent after completion.

#### 3.3.3 Physician facing translation agent

The Translation Agent operates in a non-interactive mode and is invoked only after the conversation phase is completed. Its sole responsibility is to transform the structured patient-level anamnesis into professionally formatted clinical documentation suitable for physician review.

Unlike the Conversation Agent, the Translation Agent does not perform information elicitation. Instead, it executes a unidirectional extract–transform operation, converting lay expressions into standardised medical terminology and structured clinical narrative. This includes terminological normalisation, linguistic formalisation, and organisation of content into conventional anamnesis sections (e.g., current illness, review of systems, personal and medication history). The agent is designed to be restricted from introducing new clinical facts and is intended to operate strictly on previously collected data.

The isolation of the Translation Agent serves two purposes. First, it prevents stylistic or linguistic contamination of patient interaction. Second, it allows physician-facing outputs to remain predictable, auditable, and aligned with established documentation conventions. All outputs generated by this agent are intended for human validation prior to inclusion in official medical records.

#### 3.3.4 Orchestration and data flow

Coordination between agents is managed by an external orchestration layer, which controls invocation order, state persistence, and validation checks. Figure 3 complements the high-level cognitive pipeline by explicitly illustrating agent sequencing and data flow separation. The overall data flow is strictly sequential: patient interaction precedes translation, and no feedback loop from translation to conversation is permitted within a single session. This design is intended to prevent the backward influence of professional language on patient dialogue and to preserve the fidelity of patient-reported data.

**Figure 3:**
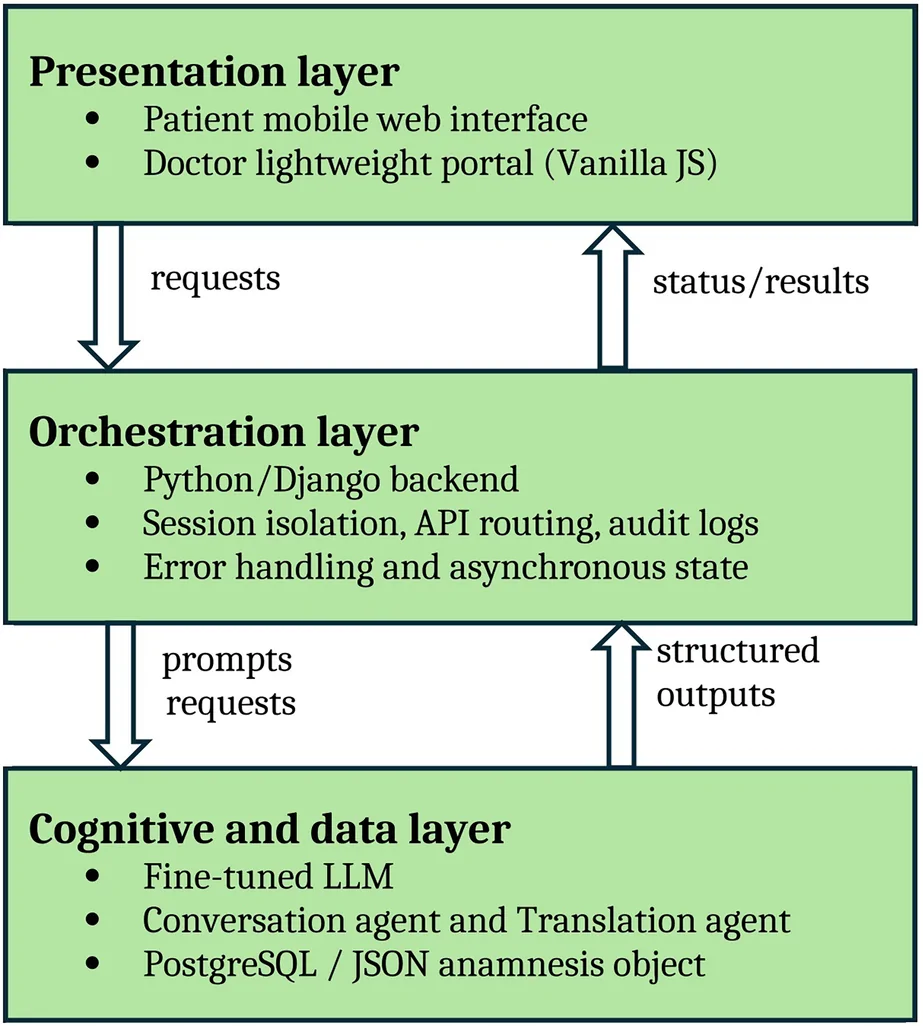
High-level architecture of Symptio.

From a safety and governance perspective, the architecture ensures that clinical authority remains with the physician. Neither agent autonomously produces diagnostic decisions or therapeutic recommendations without explicit clinician oversight. The multi-agent design thus aligns technical feasibility with medico-legal and ethical requirements for clinical decision support systems.

An external orchestration layer manages session state, agent invocation, context persistence, validation checks, and sequential data flow. The Conversation Agent remains active until framework-specific completeness criteria are satisfied. The resulting structured state is then forwarded to the Translation Agent, which transforms the collected information into physician-oriented documentation. No feedback loop from the Translation Agent to the patient-facing dialogue is permitted within a single session.

### 3.4 Knowledge representation and anamnesis framework formalisation

#### 3.4.1 Framework-driven state representation

At the core of the knowledge representation layer is an internal state-based anamnesis model, which captures selected structural elements of classical medical history taking. Each framework contributes a predefined set of dimensions that together constitute a complete anamnesis representation. These dimensions are represented as structured fields in an internal data model that is incrementally populated during patient interactions.

For symptom-focused elicitation, the SOCRATES framework defines mandatory attributes, including location, onset, character, radiation, associated symptoms, temporal pattern, modifying factors, and severity. The Review of Systems complements this vertical symptom analysis with a horizontal screening across organ systems to identify potentially relevant or overlooked complaints. The SAMPLE framework provides contextual background, including allergies, medication use, past medical history, last oral intake, and events preceding symptom onset. Collectively, these frameworks establish an explicit definition of anamnesis completeness independent of individual conversational phrasing.

To support interoperability, auditability, and agent coordination, the collected information is represented as a structured JSON-based state object. The predefined JSON-based state representation includes the chief complaint, framework-specific anamnesis attributes, contextual patient history, and interaction metadata. The state representation serves as the communication interface between the Conversation Agent and the Translation Agent.

A simplified example is shown below:

{
“chief_complaint”: “abdominal pain”,
“socrates”: {
“site”: “right lower abdomen”,
“onset”: “12 h ago”,
“character”: “sharp”,
“severity”: 7
},
“ros”: {
“nausea”: true,
“fever”: false
},
“sample”: {
“allergies”: “penicillin”,
“medications”: “none”,
“past_history”: “none”
},
“metadata”: {
“session_id”: “..”,
“completion_status”: “complete”
}
}

The complete internal representation additionally contains framework-specific attributes, confidence annotations, red-flag indicators, and audit metadata. The structured state is used exclusively for information persistence and inter-agent communication and is not presented directly to the physician.

The prototype did not employ formal JSON Schema-based validation or API-level Structured Outputs. Instead, the orchestration layer validated model outputs against the expected predefined JSON-based state representation. The complete internal state representation and corresponding validation rules are provided in Supplementary Material S3.

#### 3.4.2 Algorithmic enforcement of completeness and consistency

The formalised frameworks are not used merely as *post-hoc* classification schemas but are actively enforced during interaction. Incoming patient responses are continuously analysed and mapped onto the predefined state representation. When required fields remain empty or underspecified, the system algorithmically generates targeted follow-up prompts to obtain the missing information.

This mechanism transforms the LLM-driven dialogue from an open-ended conversation into a goal-oriented elicitation process with deterministic termination criteria. The interaction concludes only when all framework-defined requirements for the active symptom context have been satisfied, preventing premature summarisation and reducing variability across patient sessions. As a result, the output state is designed to adhere to the clinically expected structure while reducing variability associated with narrative style or patient verbosity.

Crucially, this enforcement mechanism limits model autonomy. The language model is not permitted to infer or fabricate information to satisfy missing fields. Instead, the absence of data is explicitly treated as an elicitation requirement, preserving fidelity to patient-reported content and supporting later clinical validation.

#### 3.4.3 Separation between semantic capture and medical interpretation

A key design decision in the knowledge representation layer is the strict separation between semantic capture and professional medical interpretation. The structured state produced during patient interaction retains patient-level semantics and avoids premature translation into specialised medical terminology. This intermediate representation serves as a neutral substrate that preserves the original meaning while remaining compatible with subsequent transformations.

Only after state completion is achieved does the system invoke the physician-facing Translation Agent, which operates on the structured representation rather than on raw conversational text. This staged approach is designed to ensure that linguistic normalisation and terminological formalisation occur on a controlled data structure rather than during free conversation, with the aim of reducing the risk of stylistic drift or misinterpretation.

#### 3.4.4 Transparency, auditability, and future extensibility

The explicit formalisation of anamnesis frameworks also supports transparency and auditability. Because each collected datum is associated with a corresponding framework dimension, clinicians can readily trace structured outputs back to their patient-provided origins. This traceability is essential for trust, medico-legal accountability, and alignment with human-in-the-loop validation requirements.

From an engineering perspective, the framework-driven knowledge representation enables extensibility. Additional clinical schemas or specialty-specific modules may be incorporated by defining new state dimensions and elicitation rules without altering the core conversational architecture. This modularity supports future adaptation of the platform to different outpatient contexts while preserving the underlying process-oriented design.

In the context of the present study, SOCRATES, Review of Systems, and SAMPLE are treated as explicit computational constraints governing information acquisition, completeness assessment, and information transformation. The objective is not to reproduce clinical reasoning but to formalise selected components of anamnesis collection in a machine-interpretable form.

### 3.5 Safety mechanisms and human-in-the-loop design

The deployment of large language models in clinical settings raises well-documented concerns regarding reliability, accountability, and patient safety. In the context of medical anamnesis, these concerns primarily relate to unsupported content generation, implicit clinical inference, and loss of human oversight. Symptio was designed to address these risks through a multi-layer safety design that combines architectural constraints, knowledge-level formalisation, and explicit human-in-the-loop validation. The platform is intentionally designed to support clinical documentation preparation while preserving full physician authority over interpretation and decision-making.

#### 3.5.1 Preventive risk mitigation through architectural constraints

Rather than relying solely on post-generation review to detect incorrect or unsupported outputs, Symptio prioritises preventive safety mechanisms embedded directly into system architecture. This approach aligns with current recommendations for the safety-aware deployment of LLMs in high-risk domains.

The multi-agent design described in Section 3.3 enforces role separation between data collection and medical language transformation. By isolating conversational elicitation from professional documentation generation, the system is intended to reduce context contamination and limit the risk of premature clinical interpretation during patient interaction. Each agent operates under narrowly defined success criteria and is restricted from exceeding its designated task scope. This architectural separation is designed to limit the language model's generative degrees of freedom and to mitigate the risk of unintended content creation.

In addition, the strictly sequential orchestration of agents ensures that no feedback loop exists between translation outputs and patient-facing dialogue within a single session. This prevents physician-oriented terminology from influencing patient interaction and preserves the authenticity of patient-reported information.

#### 3.5.2 Framework-bound interaction and guardrails

At the interaction level, safety is further reinforced through framework-bound questioning logic and explicit guardrails derived from clinically established anamnesis methodologies. As described in Section 3.4, SOCRATES, Review of Systems, and SAMPLE are formalised into machine-interpretable structures that define required information dimensions and completion criteria.

These frameworks function as operational guardrails, constraining the LLM's behaviour during interaction. The model is permitted to generate follow-up questions only when specific, predefined data fields remain unfilled or insufficiently specified. Conversely, the system explicitly prohibits inference, diagnosis, or assumption-based completion of missing information. The absence of data triggers structured elicitation rather than speculative generation.

This design transforms the LLM from an open-ended conversational agent into a goal-oriented information extractor constrained by deterministic clinical logic. As a result, hallucination opportunities are intended to be reduced not through after-the-fact filtering but by limiting opportunities for unsupported generation at the source.

#### 3.5.3 Transparency and auditability of LLM-mediated outputs

Transparency is a prerequisite for trust in clinical support systems. Symptio, therefore, ensures that all generated outputs remain traceable and auditable. Each structured anamnesis element can be linked back to patient-provided input and its corresponding framework dimension. This traceability enables clinicians to verify the origin and completeness of collected information and supports medico-legal accountability.

Intermediate representations generated by the Conversation Agent preserve patient-level semantics and are retained until physician review. The Translation Agent operates exclusively on this structured representation, ensuring that documentation outputs are grounded in explicit, reviewable data rather than latent conversational context. Importantly, the system does not obscure raw inputs or intermediate states, avoiding the creation of a “black box” clinical artefact.

#### 3.5.4 Human-in-the-loop validation and clinical authority

A defining design principle of Symptio is that clinical authority is never delegated to the system. The platform does not produce autonomous diagnoses, treatment recommendations, or final clinical conclusions. Instead, its outputs are explicitly positioned as pre-structured candidates for physician review.

The physician remains the final arbiter of validity, relevance, and interpretation. All system-generated documentation is subject to clinical validation, correction, or rejection prior to inclusion in official medical records. This human-in-the-loop design ensures alignment with ethical, legal, and professional standards of care and preserves clinician accountability. Figure 4 summarises this safety and validation pathway.

**Figure 4:**
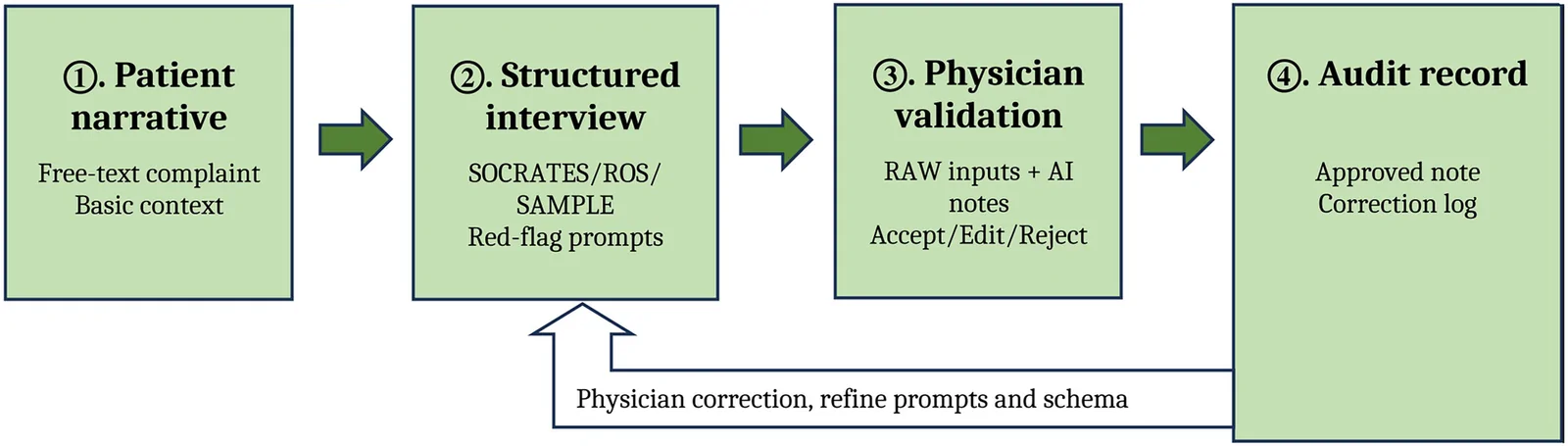
Conceptual workflow of framework-constrained information acquisition and output verification.

By relocating routine information structuring rather than clinical judgment, Symptio supports physician cognition without redefining professional responsibility boundaries. The system complements, rather than replaces, clinician expertise.

The safety mechanisms described in this section represent architectural design features of the evaluated prototype and should not be interpreted as evidence of clinical safety. Assessment of clinical safety requires prospective evaluation under real-world conditions and was outside the scope of the present proof-of-concept study.

### 3.6 Proof-of-concept implementation and evaluation setup

The present study evaluates Symptio as a proof-of-concept medical information platform, focusing on its ability to produce framework-aligned structured anamnesis representations intended for subsequent physician review within outpatient care workflows. The evaluation design intentionally prioritises methodological validity, safety, and clinical interpretability over large-scale deployment metrics, reflecting the system's early exploratory stage. The evaluation was therefore designed to assess technical feasibility and adherence to the framework rather than clinical utility, diagnostic accuracy, or healthcare outcomes.

The primary objective of the evaluation is to assess whether a process-oriented, framework-driven, multi-agent LLM architecture can support consistent and framework-consistent anamnesis representations without generating unsupported or misleading medical content. Accordingly, the evaluation does not aim to measure diagnostic accuracy, therapeutic decision quality, or patient outcomes, all of which remain explicitly within the domain of clinician responsibility.

#### 3.6.1 LLM configuration, fine-tuning, and inference settings

The evaluated prototype used a supervised fine-tuned variant of the OpenAI GPT-4.1 nano model, with gpt-4.1-nano-2025-04-14 as the base model. The same fine-tuned model was used by both the Conversation Agent and the Translation Agent and remained unchanged throughout all 100 evaluation scenarios. Fine-tuning was performed using the OpenAI fine-tuning platform and API. The training data were derived from the MediTOD medical history-taking dialogue dataset (22) and were transformed from the original dataset format into JSONL chat-message examples suitable for supervised fine-tuning. The resulting fine-tuning dataset comprised 15,000 training examples and 1,500 validation examples.

The number of training epochs, batch size, and learning-rate multiplier were not manually specified by the authors and were left at the automatic settings of the OpenAI fine-tuning service. The optimizer was likewise not manually specified by the authors and was managed internally by the fine-tuning service. No additional manual hyperparameter optimisation was performed.

During inference, sampling and generation parameters, including temperature, top_p, maximum output tokens, frequency penalty, presence penalty, and seed, were not explicitly specified in the API requests. No manual inference-parameter tuning was performed, and this configuration remained unchanged throughout all evaluation scenarios. No API-level JSON Schema-based Structured Outputs or explicit response-format constraint was used.

Agent behaviour was controlled through fixed role-specific system prompts. The complete Conversation Agent and Translation Agent system prompts are provided in Supplementary Materials S1, S2, respectively. During patient interaction, the Conversation Agent incrementally populated a predefined JSON-based state representation. Field completion was determined according to clinical relevance within the SOCRATES, ROS, and SAMPLE frameworks. Relevant fields remaining in the not_provided state, or containing insufficiently specific information, triggered targeted follow-up questioning. Conversation terminated only after all clinically relevant required fields had been resolved.

At each conversational turn, the orchestration layer supplied the Conversation Agent with the last valid persistent JSON-based state together with the patient's latest response. The agent returned a JSON turn object containing a patient-facing response and a partial state update. Following successful validation, the orchestration layer merged the returned update into the persistent state, while fields not included in the update remained unchanged. The complete turn-level interaction and state-update protocol is provided in Supplementary Materials S1, S3.

Model-generated JSON outputs were checked by the orchestration layer for syntactic validity, expected fields, data types, and permitted state values. Invalid outputs were rejected without modifying the last valid anamnesis state, and the model was requested to regenerate a corrected output. A maximum of two automatic correction attempts was permitted. If validation remained unsuccessful, the last valid state was retained and the case was flagged for technical or manual review. The complete state representation, permitted status values, and validation procedure are provided in Supplementary Material S3.

#### 3.6.2 Evaluation scope and study type

The evaluation follows a controlled, offline proof-of-concept design using simulated outpatient scenarios. This approach was chosen to allow systematic inspection of system behaviour under predefined conditions while avoiding ethical and regulatory complexities associated with live clinical deployment.

The study scope is limited to:

- outpatient primary and general medical care,
- adult, non-emergency presentation scenarios,
- structured medical anamnesis preparation prior to physician consultation.

No real-time patient care decisions, automated diagnoses, or autonomous recommendations are produced or evaluated within this study. The evaluation should be interpreted as a controlled technical assessment of information acquisition and structuring mechanisms rather than a clinical validation study.

#### 3.6.3 Scenario-based input generation

The evaluation dataset comprised 100 curated simulated outpatient scenarios representing common presentations in adult primary and general outpatient care. The scenarios were developed by members of the research team responsible for methodology, data curation, system development, and validation. Scenario construction combined manual clinical-content design with LLM-assisted generation. An LLM (GPT-5.5, reasoning effort: low) was used primarily to support the formulation of natural patient narratives and linguistic variations. All LLM-generated material was manually reviewed and, where necessary, modified by the research team before inclusion in the evaluation dataset. The scenarios were entirely synthetic and were not derived from real patient records or patient-identifiable data.

Patient inputs were formulated exclusively in natural, lay language without imposed medical terminology or predefined conversational structure. The scenarios were designed to vary in symptom presentation, language clarity, information completeness, symptom ordering, and clinical complexity.

During dataset construction, each scenario was assigned to one of five predefined clinical categories according to its dominant presenting complaint. Pain-related scenarios were defined by pain as the primary complaint irrespective of anatomical location. Respiratory scenarios were characterised by a dominant respiratory complaint, such as cough, dyspnoea, or wheezing. Gastrointestinal scenarios were characterised by dominant gastrointestinal symptoms, such as nausea, vomiting, diarrhoea, or altered bowel function; cases primarily presenting with abdominal pain were classified as pain-related. Systemic scenarios were defined by predominantly non-specific general symptoms, such as fever, fatigue, malaise, or generalised weakness. Mixed/multisymptom scenarios contained multiple co-dominant complaints involving more than one symptom domain without a single clearly dominant presenting complaint.

Scenario complexity was assessed using four predefined content-based characteristics: (1) multiple active symptoms, defined as two or more clinically relevant active symptoms; (2) involvement of two or more organ systems; (3) an incomplete or ambiguous initial presentation, defined as omission or unclear specification of information required for the relevant anamnesis and therefore requiring targeted clarification; and (4) contextual medical history, defined as the presence of at least one relevant item concerning comorbidities, medication use, allergies, or preceding events.

One point was assigned for each characteristic present. Scenarios with 0–1 points were classified as low complexity, those with 2 points as medium complexity, and those with 3–4 points as high complexity. Complexity classification was based exclusively on the predefined scenario content and was independent of the subsequent behaviour or performance of the Symptio system. The distribution of the 100 scenarios by clinical category and complexity level is summarised in Table 3.

**Table 3:** Distribution of simulated outpatient scenarios by clinical category and complexity level.

Characteristic	n	%
Clinical category		
Pain-related	28	28%
Respiratory	23	23%
Gastrointestinal	17	17%
Systemic	19	19%
Mixed/multisymptom	13	13%
Complexity level		
Low complexity	32	32%
Medium complexity	47	47%
High complexity	21	21%

#### 3.6.4 Reference standard and evaluation criteria

For each scenario, a reference-standard structured anamnesis was defined based on established clinical documentation practices and the same frameworks operationalised by the system (SOCRATES, ROS, and SAMPLE). These reference representations serve solely as comparison anchors for evaluation and are not used during system operation.

Evaluation criteria are defined at the level of information preparation and structuring, rather than diagnostic interpretation. The following qualitative and categorical dimensions are assessed:

- Completeness: whether all framework-defined anamnesis dimensions relevant to the scenario are captured.
- Correctness of information mapping: whether patient-provided content is placed into appropriate clinical categories without distortion.
- Terminological adequacy: whether professional medical terminology used in the physician-facing output accurately reflects patient-level semantics.
- Absence of unsupported content: whether the system avoids introducing symptoms, findings, or contextual information not provided or elicited from the patient.

Explicit emphasis is placed on identifying potential failure modes, such as hallucinated symptoms, misplaced emphasis, or premature clinical inference.

To provide a quantitative field-level evaluation, each clinically relevant information element generated by the system was compared with the corresponding reference-standard element. A true positive (TP) was defined as an information element that was correctly captured and assigned to the appropriate framework category. A false positive (FP) represented an unsupported information element introduced by the system, whereas a false negative (FN) represented an expected reference-standard element that was omitted or assigned to an incorrect category.

Precision was calculated as TP/(TP + FP), recall as TP/(TP + FN), and F1-score as 2 × (precision  ×  recall)/(precision + recall). The metrics were calculated separately for SOCRATES, Review of Systems, and SAMPLE. Overall micro-averaged metrics were calculated by pooling TP, FP, and FN values across all three frameworks.

#### 3.6.5 Failure-mode and clinical-severity assessment

All false-positive and false-negative information elements were subsequently subjected to a structured failure analysis. Each error was assigned to one of five failure modes: omission, unsupported addition, semantic overinterpretation, polarity error, or misclassification. An omission was defined as a reference-standard element that was absent from the generated anamnesis. An unsupported addition represented information introduced without support from the reference standard. Semantic overinterpretation referred to a stronger clinical interpretation than was supported by the available information. A polarity error represented reversal of a positive and negative finding, whereas misclassification indicated that an otherwise relevant information element was assigned to an incorrect framework or system category.

Clinical severity was classified as low, medium, or high. Low-severity errors involved clinically non-critical information and did not materially alter the overall interpretation of the anamnesis. Medium-severity errors involved clinically relevant omissions, additions, misclassifications, or polarity errors that could affect the completeness or interpretation of the prepared anamnesis and therefore required clinician correction or clarification, but did not constitute safety-critical distortion. High-severity errors were defined as errors involving safety-critical information or having the potential to materially alter clinical assessment, including clinically consequential red-flag information, critical allergy or medication information, or other errors requiring substantial revision or rejection of the prepared anamnesis. Red-flag status was assessed in the context of the complete simulated scenario rather than on the basis of an isolated symptom label.

#### 3.6.6 Qualitative output assessment

The qualitative review was performed by members of the research team with training in medical informatics and familiarity with clinical documentation standards. The review was intended to assess adherence to the framework and the consistency of outputs and should not be interpreted as independent clinical validation. Reviewers assess each generated anamnesis independently, with access to both the structured output and its corresponding patient input. The expert review focuses on:

- consistency with framework-defined information structures,
- alignment with standard documentation expectations,
- completeness and structural consistency,
- identification of any safety-relevant issues requiring correction or rejection.

Reviewers are explicitly instructed that the system output is intended as preparatory material rather than a final clinical record, reflecting the platform’s human-in-the-loop design.

#### 3.6.7 Exclusions and methodological limitations

Several evaluation dimensions are intentionally excluded from the present study. These include:

- real-world clinical deployment measurements,
- physician time savings under live conditions,
- patient satisfaction or behavioural outcomes,
- diagnostic accuracy or treatment effectiveness.

These exclusions are consistent with the proof-of-concept scope of the work and reflect the platform's positioning as a workflow-support and documentation-preparation system rather than a diagnostic tool. The study did not include independent clinical validation, inter-rater agreement analysis, or benchmarking against alternative system architectures. Future clinical studies are required to assess operational and longitudinal effects under real-world conditions.

## 4 Results

### 4.1 Completeness and structural agreement

Across the 100 simulated outpatient scenarios evaluated, Symptio produced structured anamnesis representations that generally aligned with the reference-standard framework definitions. The majority of generated anamneses showed high agreement with the reference structures on the information dimensions defined by SOCRATES, ROS, and SAMPLE.

When initial patient inputs were incomplete, the system generated targeted follow-up questions intended to populate the required dimensions or explicitly mark them as unavailable. Nevertheless, the field-level analysis identified a limited number of omitted or incorrectly categorised elements. This behaviour was observed consistently across scenarios with varying degrees of narrative clarity, supporting the robustness of the framework-driven completion logic.

Overall agreement was assessed at the level of framework field presence and correct assignment rather than narrative similarity, reflecting the system’s intended role in structured information preparation rather than text summarisation.

Overall, full structural agreement was defined at the scenario level as simultaneous full agreement across the SOCRATES, Review of Systems, and SAMPLE components. Based on this criterion, 87 of the 100 evaluated scenarios (87%) achieved full structural agreement across all three frameworks. The remaining 13 scenarios (13%) contained at least one framework-level discrepancy. At the individual framework level, full structural agreement was achieved in 96 scenarios for SOCRATES, 88 scenarios for the Review of Systems, and 93 scenarios for SAMPLE. Because framework-level discrepancies were not completely overlapping across the three frameworks, the overall all-framework agreement rate was 87%, rather than being determined solely by the lowest individual framework-level rate. Structural agreement across the evaluated frameworks is summarised in Table 4.

**Table 4:** Structural agreement between Symptio-prepared and reference-standard anamneses.

Evaluation dimension	Evaluated scenarios (n)	Full structural agreement	Scenarios with framework-level discrepancy
SOCRATES	100	96 (96%)	4 (4%)
ROS	100	88 (88%)	12 (12%)
SAMPLE	100	93 (93%)	7 (7%)
Overall anamnesis structure	100	87 (87%)	13 (13%)

The 13 scenarios that did not meet the overall full-agreement criterion contained one or more framework-level discrepancies. Across these scenarios, the field-level analysis identified 58 errors, comprising 23 false-positive and 35 false-negative elements. Clinical-severity assessment classified 30 errors as low severity and 28 as medium severity; no high-severity errors were identified. None of the 58 errors was classified as involving a red-flag indicator within the context of the corresponding simulated scenario. A detailed analysis of error types and representative examples is provided in Section 4.3 and Table 7.

The observed level of agreement provides preliminary support for the technical feasibility of framework-driven elicitation and structured information acquisition under controlled simulated conditions. In addition to the scenario-level assessment, field-level performance was evaluated across 2,400 reference-standard information elements. Of these, 2,365 elements were correctly captured, while 35 were omitted or assigned to an incorrect category. The system additionally generated 23 unsupported information elements.

For SOCRATES, precision was 99.62%, recall was 99.38%, and the F1-score was 99.50%. For the Review of Systems, precision was 98.59%, recall was 97.80%, and the F1-score was 98.19%. For SAMPLE, precision was 99.00%, recall was 98.67%, and the F1-score was 98.83%. Across all frameworks, micro-averaged precision, recall, and F1-score were 99.04%, 98.54%, and 98.79%, respectively. The detailed results are presented in Table 5. Field-level FP and FN values are reported in Table 5, while their failure modes and clinical severity are analysed in Section 4.3 and Table 7.

**Table 5:** Field-level precision, recall, and F1-score of Symptio-prepared anamneses.

Framework	TP	FP	FN	Precision	Recall	F1-score
SOCRATES	795	3	5	99.62%	99.38%	99.50%
ROS	978	14	22	98.59%	97.80%	98.19%
SAMPLE	592	6	8	99.00%	98.67%	98.83%
Overall micro-average	2365	23	35	99.04%	98.54%	98.79%

### 4.2 Terminological transformation and clinical language adequacy

Physician-facing outputs generated by the Translation Agent demonstrated consistent use of standardised medical terminology and conventional anamnesis structuring. Lay patient expressions were systematically transformed into professionally formatted formulations while preserving the original semantic intent.

Terminological equivalence was assessed using expert-aligned semantic judgment that accounted for synonymous expressions, abbreviated forms, and clinically accepted naming variants. Differences arising from synonymous terminology or alternative but equivalent clinical phrasing were not treated as errors. Minor stylistic preferences were occasionally noted, particularly when broader diagnostic descriptors could later be narrowed during direct physician–patient interaction.

Importantly, terminological transformation was performed exclusively on the structured intermediate representation produced during patient interaction. This separation was associated with consistent use of professional language in the evaluated outputs.

### 4.3 Failure analysis of false-positive and false-negative elements

A detailed failure analysis was performed for all 58 field-level errors identified across the 100 evaluated scenarios, comprising 23 false-positive and 35 false-negative elements. Framework-level distribution remained consistent with the quantitative evaluation: SOCRATES contained 3 FP and 5 FN elements, the Review of Systems contained 14 FP and 22 FN elements, and SAMPLE contained 6 FP and 8 FN elements.

Omission was the most frequent failure mode, accounting for 31 of the 58 errors. Unsupported additions accounted for 18 errors, while semantic overinterpretation occurred in four cases, polarity errors in three cases, and misclassification in two cases. The affected information fields included symptom severity, symptom associations and temporal characteristics, fever and other Review of Systems findings, medication use, allergies, past medical history, and preceding events. Thus, the observed discrepancies were not limited to stylistic or terminological variation.

Clinical-severity assessment classified 30 of the 58 errors as low severity and 28 as medium severity. No errors met the predefined high-severity criterion. The medium-severity group included, for example, omission of a reported pain severity of 8/10, omission of documented fever, omission of clinically relevant medication use, unsupported symptom additions, and polarity errors in which a positive or negative finding was reversed. Although several errors affected clinically relevant information, none was classified as involving a red-flag indicator when assessed within the full context of the corresponding simulated scenario. Representative false-positive and false-negative errors are presented in Table 7.

The absence of high-severity or red-flag-related errors in this controlled evaluation should not be interpreted as evidence of clinical safety or as evidence that such errors cannot occur in real-world use. The presence of 28 medium-severity errors demonstrates that clinically relevant discrepancies remained possible and reinforces the requirement for physician verification of all system-generated anamnesis outputs.

### 4.4 Qualitative review of generated outputs

The generated outputs were qualitatively reviewed with respect to their consistency with the predefined framework structures, completeness of information representation, terminology transformation, and suitability for subsequent human review within the proposed workflow concept.

The qualitative review indicated that the generated outputs generally adhered to the intended structural organisation defined by the SOCRATES, Review of Systems, and SAMPLE frameworks. Reviewers noted that the outputs generated by the system were predictable in structure and that preserved traceability between patient-provided information and generated documentation.

The qualitative observations summarised in Table 6 primarily concerned stylistic variation, differences in information granularity, symptom ordering preferences, and opportunities for contextual expansion. These observations should be distinguished from the field-level false-positive and false-negative errors reported in Section 4.3 and Table 7.

**Table 6:** Supplementary qualitative observations not reflected in field-level error analysis.

Observation category	Description	Clinical relevance	Typical clinician action
Stylistic variation	Use of clinically acceptable but non-preferred phrasing or broader descriptors	No impact on clinical safety or interpretability	Minor rephrasing according to individual documentation style
Granularity mismatch	Information expressed at a slightly broader or more general level than preferred by reviewer	Low; content remains clinically correct	Clarification or elaboration during consultation
Symptom ordering differences	Variability in the ordering of symptoms or history elements compared to reviewer preference	None	No action required or reordering for personal workflow
Context expansion opportunity	Patient-provided information appropriate but could benefit from contextual expansion	None	Additional questioning during encounter
Explicit uncertainty preservation	Absence of inferred data when patient input was unclear or unavailable	Positive safety feature	Clinician decision to explore further or document uncertainty

**Table 7:** Representative false-positive and false-negative errors identified in the failure analysis.

Type	Framework/field	Representative discrepancy	Failure mode	Severity
FN	SOCRATES – severity	Reported pain severity of 8/10 was omitted.	Omission	Medium
FN	ROS – fever	Measured temperature of 38.1 °C was not captured.	Omission	Medium
FN	SAMPLE – allergies	Reported seasonal pollen allergy was omitted.	Omission	Low
FP	SOCRATES – radiation	Radiation to the back was added although no radiation was reported.	Unsupported addition	Medium
FP	ROS – fever	Explicitly denied fever was represented as low-grade fever.	Polarity error	Medium
FP	SAMPLE – past medical history	Asthma was documented although the diagnosis had not been explicitly reported.	Semantic overinterpretation	Medium

Importantly, the qualitative review does not constitute evidence of clinical validity, clinical safety, or clinical effectiveness. The dedicated failure analysis identified 58 field-level discrepancies, including 28 medium-severity errors, demonstrating that clinically relevant omissions and unsupported additions remained possible. Consequently, all generated outputs require physician verification prior to use in clinical documentation.

The qualitative findings should therefore be interpreted as evidence that the proposed architecture can generate framework-aligned structured representations under controlled simulated conditions rather than as evidence of readiness for clinical deployment.

### 4.5 Qualitative observations and limitations

Several qualitative observations emerged during evaluation. Scenarios involving multiple concurrent complaints required longer interaction sequences but did not result in structural degradation. Conversely, highly focused complaints were resolved efficiently with minimal follow-up.

The evaluation also highlighted limitations inherent to the proof-of-concept design. Results are based on simulated scenarios and controlled expert review rather than real-world clinical deployment. Consequently, findings should be interpreted as evidence of technical feasibility under controlled simulated conditions rather than as validation of clinical safety or operational impact.

The evaluation results indicate that the prototype generated structured anamnesis representations from unstructured patient narratives with high field-level performance while maintaining physician oversight. High field-level performance was observed within the evaluated framework-driven multi-agent architecture incorporating human-in-the-loop validation. These findings support the feasibility of relocating the structuring of routine anamnesis to the pre-consultation phase in outpatient care.

The study was not designed as a comparative benchmark of foundation models; therefore, the influence of alternative LLMs on the quality of anamnesis was not assessed.

### 4.6 Prototype implementation

The proof-of-concept prototype was implemented as a distributed web-based system. The orchestration layer and application logic were implemented using the Django framework, while structured state persistence was supported through PostgreSQL. Patient and physician interfaces communicated with the backend through HTTPS-based API calls. Information exchange between system components relied on predefined JSON structures. The physician-facing interface was implemented as a lightweight web client to facilitate future integration with existing clinical information systems.

The architecture employs two role-specialised agents operating sequentially within the same workflow. The Conversation Agent is responsible for framework-driven information elicitation and operates under constraints derived from the SOCRATES, Review of Systems, and SAMPLE methodologies. The Translation Agent receives only the completed structured state and transforms the collected information into clinician-oriented medical documentation. The Translation Agent is restricted to transforming previously collected information and is not permitted to introduce new clinical facts.

Dialogue completion is determined by framework-level completeness checks. Follow-up questions are generated only for missing or insufficiently specified dimensions. The conversation terminates when all required fields are populated or explicitly marked as unavailable.

Session state is maintained throughout the interaction by the orchestration layer, which continuously evaluates framework completeness and stores intermediate results. Once the elicitation phase is completed, the structured state is passed to the Translation Agent through a JSON-based interface, ensuring separation between patient-facing dialogue and physician-facing documentation generation.

### 4.7 Regulatory and governance considerations

While the present study focuses on the technical feasibility of a framework-driven multi-agent architecture for structured anamnesis preparation, future clinical deployment would require consideration of applicable regulatory and governance frameworks.

Within the European Union, AI-enabled healthcare systems may fall under the scope of the Medical Device Regulation [MDR, Regulation (EU) 2017/745] and the Artificial Intelligence Act [Regulation (EU) 2024/1689], depending on their intended use, level of autonomy, and integration into clinical workflows. These frameworks emphasise risk management, transparency, data governance, human oversight, accountability, and post-market monitoring requirements. Similar regulatory expectations are reflected in FDA guidance for AI-enabled medical technologies.

The architecture presented in this study was intentionally designed around human-in-the-loop principles. Physicians remain responsible for reviewing, validating, modifying, or rejecting system-generated outputs before any information is incorporated into clinical documentation. Consequently, the platform is intended to support information collection and documentation preparation rather than autonomous clinical decision-making.

As the current work represents a proof-of-concept evaluation performed under controlled conditions, no claims regarding regulatory compliance, certification, or clinical readiness are made. Assessing regulatory pathways, governance requirements, privacy protection mechanisms, and compliance with applicable healthcare AI regulations is an important direction for future research and development.

It is important to distinguish between safety considerations related to LLM-mediated information processing and those associated with real-world clinical deployment. The present study focuses primarily on architecture-level safeguards, including framework-driven information elicitation, agent role separation, transparency, and physician validation mechanisms, intended to reduce the generation of unsupported content. Broader safety requirements associated with operational deployment, such as cybersecurity, data protection, privacy management, regulatory compliance, access control, and post-market governance, were not evaluated within the scope of this proof-of-concept study and will require dedicated investigation prior to any clinical implementation.

## 5 Discussion

This study explored the technical feasibility of operationalising established anamnesis frameworks (SOCRATES, ROS, and SAMPLE) as machine-interpretable workflow constraints within a process-oriented LLM architecture. Rather than evaluating diagnostic capabilities or clinical performance, the study focused on whether structured anamnesis acquisition could be represented as a constrained information-engineering workflow operating prior to the clinical encounter.

The results indicate that the proposed framework-driven architecture can transform unstructured patient narratives into structured representations aligned with predefined anamnesis frameworks. Full structural agreement across all three frameworks was observed in 87% of the evaluated scenarios, while framework-specific agreement ranged from 88% for the Review of Systems to 96% for SOCRATES. The observed structural agreement indicates that framework-driven information acquisition can be implemented in a technically consistent manner under controlled simulated conditions. The results suggest that explicit representation of SOCRATES, Review of Systems, and SAMPLE dimensions can reduce variability associated with free-text patient narratives and support reproducible information structuring.

Field-level evaluation demonstrated high quantitative performance across all three clinical frameworks. The overall micro-averaged precision of 99.04% indicates that unsupported information was generated infrequently, while the recall of 98.54% reflects a low proportion of omitted or incorrectly categorized reference-standard elements. The resulting micro-averaged F1-score of 98.79% demonstrates a strong balance between precision and recall under the controlled simulated evaluation conditions. Nevertheless, the identification of 23 false-positive and 35 false-negative elements confirms that the system does not eliminate unsupported generation or information omission and that physician validation remains necessary.

A key contribution of this work lies not in the development of a novel language-model architecture but in the formalisation of established anamnesis methodologies as explicit computational constraints. The role-specialised architecture was adopted as a practical mechanism for controlling information transformation within the evaluated workflow. Whether this specific decomposition is necessary or superior to alternative designs remains to be established through comparative studies. Although the present study was not designed to compare multi-agent and alternative implementations, the observed behaviour suggests that explicit task separation may provide a practical mechanism for controlling information transformation within structured anamnesis workflows.

No identified field-level error was classified as high severity or as involving a red-flag indicator within the controlled simulated scenarios. However, the occurrence of 28 medium-severity errors, including omissions of clinically relevant information and unsupported additions, demonstrates that the system cannot be assumed to produce clinically complete or error-free outputs. These findings reinforce the intended positioning of Symptio as a documentation-preparation tool requiring physician verification rather than as an autonomous clinical system.

Most existing LLM-based medical systems optimise functionality at the level of the clinical encounter, such as ambient documentation, summarisation, or on-demand decision support. In contrast, this work adopts a process-oriented perspective, explicitly modelling when and by whom information is collected and transformed.

The results suggest that relocating routine anamnesis structuring to a pre-consultation phase is both feasible and compatible with safety-oriented design principles. This temporal reallocation does not diminish the physician–patient interaction but rather enters it with better-prepared contextual information. Such re-engineering of clinical workflows represents an underexplored dimension of LLM application in healthcare and may offer complementary benefits to encounter-centric documentation tools.

Several limitations should be acknowledged. First, the evaluation is based on simulated outpatient scenarios and expert review rather than on deployment in live clinical environments. While this design enables controlled inspection of system behaviour, real-world factors such as patient interaction variability, time pressure, and dynamics of clinical context were not assessed. The present study was designed as a proof of concept to establish the technical feasibility of framework-driven anamnesis acquisition. Comparative evaluation of architectural variants, including single-agent configurations, was outside the scope of this work. However, the observed performance (87% full structural agreement, 98.79% micro-averaged F1) demonstrates that the proposed architecture can consistently structure information in a framework-aligned manner under controlled conditions. Systematic ablation studies comparing alternative implementations represent an important direction for future research.

Second, the study focuses exclusively on outpatient primary and general medical care. Although the underlying framework-driven approach is modular, additional validation would be required to assess applicability in specialised disciplines or emergency settings, where time constraints and information priorities differ substantially.

Furthermore, the study did not evaluate operational safety aspects of clinical deployment, including cybersecurity, personal data protection, privacy management, and regulatory compliance requirements.

Finally, this work does not quantify operational outcomes, such as reductions in consultation time or changes in clinician workload. These metrics are intentionally excluded at the proof-of-concept stage and constitute important directions for future clinical studies.

Although the evaluation included 100 simulated outpatient scenarios, the study remains a controlled proof-of-concept based exclusively on simulated inputs, which limits statistical generalisability and prevents direct inference to real-world clinical settings. The study was also not designed as a comparative benchmark against alternative LLMs or conventional anamnesis collection approaches. Finally, although unsupported information elements were infrequent, their occurrence confirms that the system cannot be considered hallucination-free, and broader clinical settings may reveal additional failure modes.

An additional limitation is that the study does not provide evidence regarding the comparative effectiveness of the proposed architecture. No baseline comparisons, ablation studies, or alternative workflow implementations were evaluated. We did not evaluate the system against alternative implementations, such as a single-agent system, a model without fine-tuning, or a system without explicit framework constraints. This comparative study will be implemented in future experimental evaluations. Furthermore, the study does not constitute clinical validation and provides no evidence regarding diagnostic performance, patient outcomes, or clinical effectiveness. Consequently, the present results should be interpreted as evidence of technical feasibility rather than evidence that the proposed architecture is superior to other possible implementations.

Future studies should also examine regulatory, governance, and compliance requirements associated with the deployment of AI-assisted anamnesis systems in clinical practice. The findings of this study highlight several avenues for future work. Prospective clinical evaluations are needed to assess real-world adoption, physician satisfaction, and patient experience. Integration with electronic health record systems and exploration of speciality-specific framework extensions represent additional technical directions.

More broadly, this work suggests that meaningful and safe application of LLMs in healthcare may depend less on incremental gains in model performance and more on careful system design, explicit knowledge formalisation, and process-aware integration. As generative models continue to advance, such design principles may prove essential for translating technical capability into clinically responsible practice.

In summary, this study demonstrates the technical feasibility of implementing framework-driven anamnesis acquisition within a process-oriented LLM architecture operating under human supervision. The principal contribution lies in the formalisation of established anamnesis methodologies as explicit computational constraints and in the exploration of workflow-oriented knowledge representation rather than in the development of a clinically validated decision-support system. The findings suggest that framework-driven, process-oriented architectures represent a promising direction for future investigation of LLM-assisted anamnesis preparation. Whether such approaches improve clinical workflows, physician efficiency, documentation quality, or patient outcomes remains to be established through prospective clinical studies.

## 6 Conclusions

This work presents Symptio, a proof-of-concept medical information platform that demonstrates how established anamnesis methodologies can be operationalised as machine-interpretable workflow constraints within a process-oriented LLM architecture. By relocating structured anamnesis preparation to a pre-consultation phase and grounding interaction logic in clinically established frameworks, the platform is intended to address a persistent mismatch between unstructured patient narratives and structured clinical documentation requirements.

The presented results demonstrate the technical feasibility of representing structured anamnesis acquisition as a framework-driven information-engineering workflow operating under explicit human supervision. Simultaneous full structural agreement across SOCRATES, Review of Systems, and SAMPLE was achieved in 87% of the evaluated scenarios. Together with micro-averaged precision of 99.04%, recall of 98.54%, and F1-score of 98.79%, these findings are consistent with the technical feasibility of the proposed framework-driven approach under controlled simulated conditions and explicit human-in-the-loop oversight. Detailed analysis of the identified field-level errors showed that information-level discrepancies remained possible despite the applied architectural constraints and framework-driven elicitation logic. These findings further support the necessity of physician verification of all system-generated outputs. Importantly, the system is positioned as an assistive tool for documentation preparation and does not perform autonomous diagnosis or clinical decision-making.

The principal contribution of this work lies not in proposing a novel language-model architecture, but in exploring how established clinical anamnesis frameworks can be translated into explicit computational constraints within a complete, process-oriented workflow. The presented proof-of-concept demonstrates the technical feasibility of this approach. Future work, including comparative ablation studies, is required to determine the individual contribution of specific architectural components, such as the multi-agent separation, to the overall system performance. Accordingly, the proposed platform should be interpreted as an exploration of workflow-oriented knowledge representation and process design rather than as a clinically validated decision-support solution.

Future research should focus on prospective clinical validation in real outpatient settings, including the assessment of physician acceptance, patient usability, workflow integration, consultation efficiency, and clinical effectiveness. Additional studies should evaluate robustness across different foundation models, compare framework-driven architectures with alternative implementations, conduct formal inter-rater agreement analyses, and benchmark against conventional approaches to anamnesis collection. Further work is also needed to investigate integration with electronic health record systems, adaptation to speciality-specific anamnesis frameworks, and regulatory, governance, privacy, and data-protection requirements relevant to clinical deployment.

## Data Availability

The datasets analyzed in this study are available from the corresponding author upon reasonable request.
