# Supplementary material for: A multi-agent large language model (LLM) platform for structured medical anamnesis in outpatient care
Source: Front Digit Health. 2026 Sep 30;8:1882397. doi: 10.3389/fdgth.2026.1882397 (PMC13662122; doi:10.3389/fdgth.2026.1882397)
Supplement: Supplementary file 1 [file Supplementaryfile1.docx]

Supplementary Material

# 1 Supplementary Material S1. Conversation Agent System Prompt

The prompt below corresponds to the complete system-level instruction used throughout the evaluation. The following system prompt was used for the Conversation Agent throughout the evaluation:

*You are a medical anamnesis assistant. You are invoked once for each patient turn. Use the current validated anamnesis state together with the patient's latest response to collect and update the structured medical history according to the SOCRATES, Review of Systems (ROS), and SAMPLE frameworks.*

*Return only one valid JSON object containing response_to_patient, state_update, and metadata.completion_status. In state_update, include only information explicitly provided by the patient and only fields whose status or value needs to be updated. Do not infer or invent missing information, and leave all other fields unchanged. For general anamnesis fields, use known, unknown, not_provided, or not_applicable; for individual ROS findings, use present, absent, unknown, or not_provided.*

*In response_to_patient, ask one clear and patient-friendly question addressing the most relevant unresolved field, without repeating questions that have already been answered. When all clinically relevant fields are resolved, set metadata.completion_status to complete and provide a brief completion message instead of another question. Do not provide diagnoses or treatment recommendations.*

# 2 Supplementary Material S2. Translation Agent System Prompt

The prompt below corresponds to the complete system-level instruction used throughout the evaluation. The following system prompt was used for the Translation Agent throughout the evaluation:

*You are a medical documentation assistant. Convert the structured patient anamnesis into concise and professional medical terminology suitable for physician review. Preserve the original meaning, negations, and uncertainty, and do not add new symptoms, diagnoses, assumptions, or treatment recommendations.*

# 3 Supplementary Material S3. Internal JSON-Based State Representation and Validation Rules

The evaluated prototype did not employ a formal JSON Schema or API-level JSON Schema-based Structured Outputs. Instead, the system maintained anamnesis information using the following predefined JSON-based state representation:

{

"chief_complaint": {

"status": "not_provided",

"value": null,

"confidence": null

},

"socrates": {

"site": {

"status": "not_provided",

"value": null,

"confidence": null

},

"onset": {

"status": "not_provided",

"value": null,

"confidence": null

},

"character": {

"status": "not_provided",

"value": null,

"confidence": null

},

"radiation": {

"status": "not_provided",

"value": null,

"confidence": null

},

"associations": {

"status": "not_provided",

"values": [],

"confidence": null

},

"time_course": {

"status": "not_provided",

"value": null,

"confidence": null

},

"exacerbating_factors": {

"status": "not_provided",

"values": [],

"confidence": null

},

"relieving_factors": {

"status": "not_provided",

"values": [],

"confidence": null

},

"severity": {

"status": "not_provided",

"value": null,

"confidence": null

}

},

"ros": [

{

"system": "",

"status": "not_provided",

"findings": [

{

"finding": "",

"status": "not_provided",

"confidence": null

}

]

}

],

"sample": {

"symptoms": {

"status": "not_provided",

"values": [],

"confidence": null

},

"allergies": {

"status": "not_provided",

"values": [],

"confidence": null

},

"medications": {

"status": "not_provided",

"values": [],

"confidence": null

},

"past_medical_history": {

"status": "not_provided",

"values": [],

"confidence": null

},

"last_oral_intake": {

"status": "not_provided",

"value": null,

"confidence": null

},

"events_preceding": {

"status": "not_provided",

"value": null,

"confidence": null

}

},

"red_flags": [],

"metadata": {

"session_id": "",

"completion_status": "in_progress"

}

}

Confidence values were reserved for future extensions and were not used during the present evaluation.

# State and completion rules

For general anamnesis fields, the permitted states were known, unknown, not_provided, and not_applicable. known indicated that the required information had been provided by the patient. unknown indicated that the patient had been asked but was unable to provide the information. not_provided indicated that the information had not yet been obtained, while not_applicable indicated that the field was not clinically relevant to the specific case.

For individual Review of Systems findings, the permitted states were present, absent, unknown, and not_provided. This distinction prevented missing information from being interpreted automatically as a negative finding.

Field requirements were determined according to clinical relevance rather than through a universally fixed mandatory/optional designation. All fields required for completion of the clinically relevant SOCRATES, ROS, and SAMPLE components were treated as required for the corresponding case.

After each patient response, the current state of the relevant fields was evaluated. A field required additional elicitation when it remained not_provided or when the available patient response was insufficiently specific to populate it reliably. Missing information was not inferred solely for the purpose of completing the state.

The Conversation Agent terminated when all clinically relevant required fields had been resolved. General anamnesis fields were considered resolved when their status was known, unknown, or not_applicable. Individual ROS findings were considered resolved when classified as present, absent, or unknown.

**Turn-level interaction and state-update protocol**
At each conversational turn, the orchestration layer supplied the Conversation Agent with the last valid persistent JSON-based anamnesis state and the patient’s latest response. The model returned a single JSON turn object containing response_to_patient, state_update, and metadata.completion_status. The state_update object represented a partial update of the persistent state; fields omitted from the update remained unchanged.

Before the update was applied, the orchestration layer validated the returned object for JSON syntax, permitted field names, expected data types, and permitted status values. Following successful validation, the update was merged into the last valid persistent state. The session_id was maintained by the orchestration layer rather than generated by the language model. If validation failed, the persistent state remained unchanged and the correction-and-retry procedure described below was applied.

The response_to_patient value was used as the next patient-facing message. When metadata.completion_status was set to complete, no further elicitation question was generated and the complete persistent state was forwarded to the Translation Agent.

# Validation and retry procedure

Model-generated JSON outputs were checked for syntactic JSON validity, presence of expected fields, expected data types, permitted status values, and consistency with the predefined JSON-based state representation.

An invalid output was rejected and was not used to update the current anamnesis state. The last valid state was preserved and the model received a correction request based on the detected validation error. A maximum of two automatic correction attempts was permitted.

If the output remained invalid after these attempts, the last valid state was retained and the corresponding case was flagged for technical or manual review.

No formal validation against the JSON Schema standard was used in the evaluated prototype.
